# Simultaneous Catecholaminergic Polymorphic Ventricular Tachycardia and Long QT Syndrome Gene Mutations

**DOI:** 10.7759/cureus.19195

**Published:** 2021-11-01

**Authors:** Muhddesa Lakhana, James McGee, Blessen George, William Whang, Lawrence Kanner, Won Jun Park

**Affiliations:** 1 Internal Medicine, Mount Sinai South Nassau, Oceanside, USA; 2 Cardiology, Icahn School of Medicine at Mount Sinai, New York, USA; 3 Cardiology, Mount Sinai South Nassau, Oceanside, USA

**Keywords:** internal medicine-cardiology, cardiology research, cardiovascular genetics, long qt, cpvt

## Abstract

Genetic channelopathies can predispose individuals to life-threatening arrhythmias. Two such channelopathies are long QT syndrome (LQTS) and catecholaminergic polymorphic ventricular tachycardia (CPVT). To the best of our knowledge, we present the first case of LQTS with novel combined genetic mutations of KCNH2 and cardiac ryanodine receptor (RYR2) genes.

## Introduction

The cardiac system relies on the proper functioning of ion channels for the propagation of cardiac action potentials. Genetic channelopathies can predispose individuals to life-threatening arrhythmias [1]. Two such channelopathies are long QT syndrome (LQTS) and catecholaminergic polymorphic ventricular tachycardia (CPVT).

LQTS is the most common genetic arrhythmia syndrome, and it is associated with sudden death in otherwise healthy young people [2]. Since the first reported cases in 1975 [3], the phenomenon has been extensively studied and the understanding of its mechanism has evolved over time. While multiple mutations have been implicated in the pathogenesis of LQTS, KCNQ1 (LQT1), KCNH2 (LQT2), and SCN5A (LQT3) are the most common LQTS gene mutations, accounting for approximately 90% of all genotype-positive cases [4]. Aside from congenital causes for LQTS, other causes should also be explored when a patient presents with a prolonged QT interval. Additional causes include medications and electrolyte imbalance.

CPVT is a rare condition characterized by syncope or cardiac arrest after physical exercise or emotional stress in individuals without structural cardiac abnormalities. CPVT most commonly results from mutations in many genes, two of the most common ones being the cardiac ryanodine receptor (RYR2) gene and the calsequestrin 2 (CASQ2) gene [5,6]. 

To the best of our knowledge, we present the first case of LQTS with novel combined genetic mutation of KCNH2 and RYR2 genes as well as other precipitating factors.

## Case presentation

**Figure 1:**
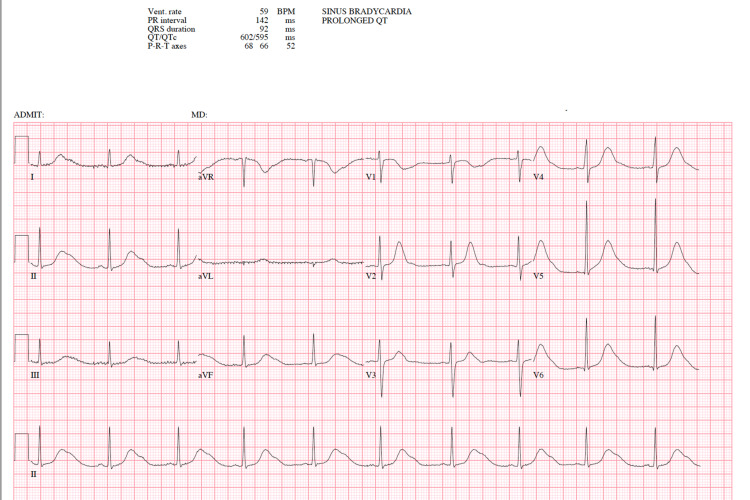
EKG 1 Sinus bradycardia. Wide-notched T waves with prolonged QTc interval EKG: electrocardiogram

A 25-year-old African American female with a past medical history of cannabis hyperemesis syndrome, anxiety, and depression on escitalopram (Lexapro) presented to our emergency room for two days of abdominal pain and intractable vomiting with nausea relieved by hot showers. She reported having a similar episode five months prior, which had required hospitalization for 21 days for the management of electrolyte imbalance. At that time, she reported having multiple arrhythmias and cardiac MRI but stated that her workup had been negative. Her arrhythmias had been attributed to hypokalemia. Since that hospitalization, the patient reported losing 30lb and had not followed up as an outpatient for her arrhythmia. Physical exam revealed normal heart sounds, no murmurs, no evidence of jugular venous distension, and clear lung sounds bilaterally. In the emergency room, labs were notable for hypokalemia (3.4 mEq/L), hypophosphatemia (2.1 mg/dL), and normal serum magnesium (1.9 mg/dL). Also, emergency room electrocardiogram (EKG) revealed sinus bradycardia, wide-notched T waves, and prolonged QT ranging from 550 to 800 msec; QTc was 595 msec (Figure 1).

**Figure 2:**
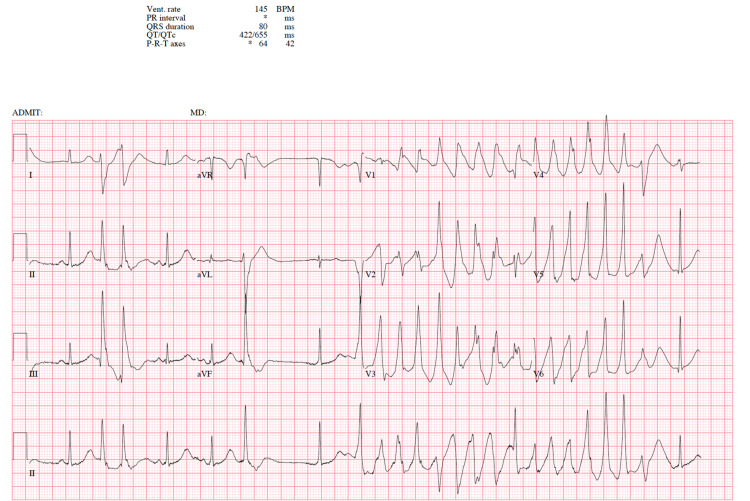
EKG 2 Torsades de pointes EKG: electrocardiogram

The urine drug screen was positive for tetrahydrocannabinol (THC). The echocardiogram revealed an ejection fraction (EF) of 50-55%, normal right ventricular systolic function, and no significant valvular disease. In the ED, the patient was started on ondansetron for nausea and vomiting. On the medical floor, her serum potassium fell to 2.7 mEq/L, and she developed premature ventricular contractions (PVCs) and subsequently an episode of torsades de pointes on telemetry after receiving ondansetron, a QT-prolonging medication (Figure 2).

**Figure 3:**
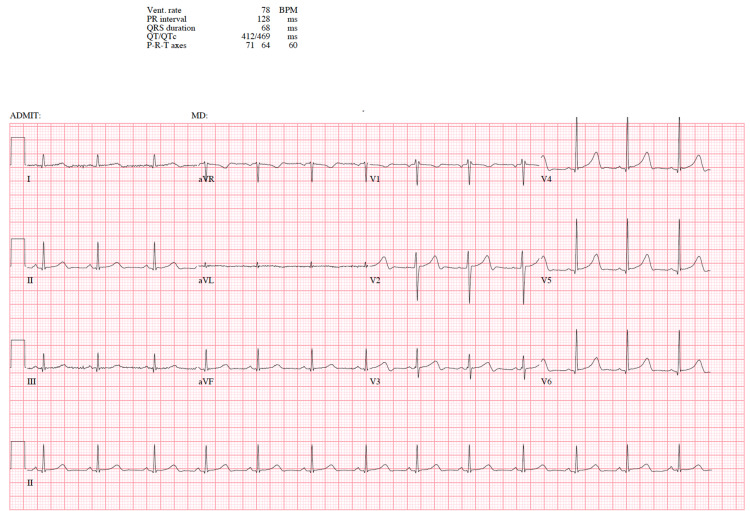
EKG 3 Normal sinus rhythm EKG: electrocardiogram

The arrhythmia resolved spontaneously; however, she was transferred to ICU for further monitoring. Once her electrolyte imbalance resolved and QT-prolonging medications, ondansetron, and escitalopram were discontinued, her EKG returned to the baseline of normal sinus rhythm (Figure 3).

Subsequent genetic testing using Invitae Diagnostic (Invitae, San Francisco, CA) ultimately revealed two uncertain variants of the KCNH2 and RYR2 genes with c.2692+7c>T and c.3932C>T variants, respectively.

The patient was started on propranolol per Electrophysiology and a loop recorder was placed prior to discharge.

## Discussion

LQTS is the most common genetic arrhythmia syndrome, which is associated with sudden death in otherwise healthy young people [2]. Many individuals are carriers of these genetic mutations but are often asymptomatic. Prolonged QT interval (>440 msec in males, >460 msec in females) increases the risk for arrhythmias such as torsades de pointes, often causative of syncope, and ventricular fibrillation, possibly fatal. Common presentations for LQTS include palpitations, arrhythmia, syncope, and cardiac arrest. Ultimately, the diagnosis is clinical, but genetic testing is confirmatory in the setting of long QT intervals. The Schwartz scoring system is often used to guide the diagnosis of LQTS, but other causes of prolonged QT interval should also be evaluated. Acquired causes of prolonged QT interval include electrolyte abnormalities including hypokalemia and hypocalcemia, endocrine abnormalities such as hypothyroidism, and medications such as dofetilide, sotalol, haloperidol, methadone, and pentamidine [4]. Among patients with LQTS, the QTc duration is the most useful risk stratifier.

CPVT is characterized by syncope or cardiac arrest after increased stress to the cardiac system, which can result from physical exercise or emotional stress. The diagnosis of CPVT is established in either the presence of a structurally normal heart with normal resting EKG and catecholamine-induced bidirectional or polymorphic ventricular tachycardia, or in individuals who have the genes for CPVT [5]. CPVT most commonly results from mutations in the RYR2 and CASQ2 genes [6,7]. Both these genes are associated with the release of calcium. When stressed, EKG findings in individuals with CPVT consist of bidirectional or polymorphic ventricular tachycardias, which can either resolve on its own or degenerate into ventricular fibrillation and lead to sudden death if not treated immediately [8].

At baseline, the patient discussed in this case had an EKG with a normal QT interval (Figure 3). On initial presentation to the ED, the patient was on Lexapro, a known QT-prolonging medication. She also had mild electrolyte abnormality (potassium: 3.4 mEq/L) and we appreciated a prolonged QT interval with notched T waves on EKG. At that time, it was unclear whether her EKG changes were due to an underlying genetic channelopathy or due to her medication and electrolyte imbalance. Once the patient received a few doses of ondansetron, another known QT-prolonging medication, and her potassium further dropped, she developed PVCs and torsades de pointes (Figure 2). The arrhythmia spontaneously resolved. Her EKG returned to normal sinus rhythm (Figure 3) once the QT-prolonging agents were removed and her electrolytes were corrected. Due to a concern for recurrent arrhythmias, genetic testing was performed and revealed KCNH2 and RYR2 gene mutations. Upon further literature search, we have found no other case in which a patient had provoked QT prolongation with underlying mutations in both KCNH2 and RYR2 genes. The management of CPVT is similar to that of LQTS. An implantable cardioverter-defibrillator (ICD) may be indicated in high-risk individuals. Cornerstones of management also include avoidance of sports activities and intense physical exertion as well as beta-blockade.

An implantable loop recorder was placed during the admission and the patient was started on propranolol upon discharge. During subsequent outpatient visits with cardiology and electrophysiology, the patient reported improvement in her condition. No arrhythmia has been observed on the loop recorder and the patient continues to take propranolol.

## Conclusions

We encountered a rare case of a patient with gene polymorphism for LQT and CPVT channelopathies. Electrolyte imbalance, medications, and the underlying genetic mutations likely contributed to her torsade de pointes. Since there is no other reported case of polymorphism of these two channelopathies, our patient was started on propranolol, and a loop recorder was placed to record any arrhythmias. Furthermore, she was advised to avoid any medications, and substances that could increase her heart rate, prolong QT, and lead to arrhythmia. Moving forward, she has been advised to consult with her cardiologist before taking any over-the-counter or prescription medications.

Despite these steps, we are unsure as to what this means for the patient in the future. We have not observed any episodes of ventricular tachycardia indicating CPVT on her loop recorder or during the admission, which raises the question as to what are the possible effects of this gene polymorphism on the patient. If she chooses to become pregnant, we are unsure what the consequence of both KCNH2 and RYR2 gene polymorphism would be for the patient and her offspring. Genetic counseling has been offered to the patient; however, we do not have enough data and literature on this topic to understand the long-term consequences. Going forward, more research needs to be conducted in this field.
